# Expression and function of lysophosphatidic acid receptors (LPARs) 1 and 3 in human hepatic cancer progenitor cells

**DOI:** 10.18632/oncotarget.6696

**Published:** 2015-12-20

**Authors:** Valentina Zuckerman, Eugene Sokolov, Jacob H. Swet, William A. Ahrens, Victor Showlater, David A. Iannitti, Iain H. Mckillop

**Affiliations:** ^1^ Department of Surgery, Carolinas Medical Center, Charlotte, NC, USA 28203; ^2^ Department of Pathology, Carolinas Medical Center, Charlotte, NC, USA 28203

**Keywords:** lysophosphatidic acid (LPA), hepatocellular carcinoma (HCC), SKHep1, cell migration, Gi-protein

## Abstract

Hepatocellular carcinoma (HCC) is the most common primary cancer of the liver and is characterized by rapid tumor expansion and metastasis. Lysophosphatidic acid (LPA) signaling, *via* LPA receptors 1–6 (LPARs1–6), regulates diverse cell functions including motility, migration, and proliferation, yet the role of LPARs in hepatic tumor pathology is poorly understood. We sought to determine the expression and function of endothelial differentiation gene (EDG) LPARs (LPAR1–3) in human HCC and complimentary *in vitro* models. Human HCC were characterized by significantly elevated LPAR1/LPAR3 expression in the microenvironment between the tumor and non-tumor liver (NTL), a finding mirrored in human SKHep1 cells. Analysis of human tissue and human hepatic tumor cells *in vitro* revealed cells that express LPAR3 (HCC-NTL margin *in vivo* and SKHep1 *in vitro*) also express cancer stem cell markers in the absence of hepatocyte markers. Treatment of SKHep1 cells with exogenous LPA led to significantly increased cell motility but not proliferation. Using pharmacological agents and cells transfected to knock-down LPAR1 or LPAR3 demonstrated LPA-dependent cell migration occurs *via* an LPAR3-Gi-ERK-pathway independent of LPAR1. These data suggest cells that stain positive for both LPAR3 and cancer stem cell markers are distinct from the tumor mass *per se*, and may mediate tumor invasiveness/expansion via LPA-LPAR3 signaling.

## INTRODUCTION

Hepatocellular carcinoma (HCC) is the most common primary tumor of the liver and accounts for more than 750,000 deaths/yr. In most instances HCC arises following exposure to known risk factors, the most common being viral hepatitis infection (HBV/HCV), prolonged, heavy alcohol use, aflatoxin ingestion and obesity [1]. Mainstay therapies for HCC rely on surgical intervention (resection, ablation, and/or transplant) with alternatives being limited in scope and efficacy [1, 2]. Outcomes for HCC patients are often compromised by advanced tumor stage, impaired liver function as a result of underlying cirrhosis, and the presence of extra- and/or intra-hepatic metastasis. As a result, the prognosis for HCC patients remains bleak, survival times of 7–12 months following diagnosis being typical [1, 2].

Lysophosphatidic acid (LPA) is a small phospholipid molecule comprising of a phosphate group, glycerol backbone, and single fatty acid chain. Biologically, LPA exists in many structural variants based on degree of saturation, position of the C = C (when present), and number of carbons in the fatty acid chain [3, 4]. Synthesis of LPA occurs from membrane phospholipids, primarily *via* lysophospholipase D (autotaxin) and lysophospholipase A1β [3, 5, 6]. Following synthesis LPA regulates diverse cell functions across a range of cell types including proliferation, survival, and migration [3]. To do so LPA acts as an extracellular agonist binding to G-protein-coupled LPA receptors (LPARs) of which 6 have been characterized to date (LPARs1–6) [3, 7, 8]. Each receptor differs in cell/tissue distribution, agonist-binding profile, and downstream intracellular signaling pathway(s) regulated following activation. Based on structural and phylogenetic homology LPARs can be divided into two major sub-groups: the endothelial differentiation gene (EDG) sub-family (LPARs 1–3), and the non-EDG sub-family (LPARs 4–6) [7].

Given LPA's capability to regulate diverse basic cell functions, it is unsurprising that LPA signaling is also exploited by malignant cells and is altered in many cancers. This aberrant regulation is evident at various levels including escalation in LPA synthesis, changes in circulating LPA profile, and altered LPAR expression profiles [9–11], and occurs in various cancers including ovarian [12], breast [13], colon [14], and pancreatic tumors [15, 16]. Unlike other organs the role of LPAR signaling in normal liver function has proven more ambiguous due to the [relative] lack of previously well-characterized LPARs (LPARs 1–5) in healthy liver/hepatocytes [4, 17–19]. Analysis of serum samples report elevated LPA levels in HCC patients [10, 20] and animal models of liver disease [21]. Circulating LPA, and changes in LPA isoform composition, are also indicated as potential markers of HCV patient progression to HCC [21], and as early markers of HCC development [9, 10]. Within cirrhotic patients, LPA signaling is linked with hepatic stellate cell activation [22, 23] and tumor-derived LPA has been reported to be central to peritumoral fibroblast recruitment and transdifferentiation into myofibroblasts and accelerated tumor progression [20].

Studies by our group and others now report LPAR6, the most recently characterized LPAR subtype [24, 25], is expressed in normal liver/hepatocytes, and is significantly elevated in human HCC [26, 27] and regenerating rodent liver [28]. During the course of these studies we reported LPAR1 and LPAR3 expression was increased in a subset of human HCC and cirrhotic non-tumor liver (NTL) compared to liver from non-tumor burdened patients [27]. In the current study we further analyzed EDG-LPAR (LPARs1–3) expression and localization in human HCC specimens. These studies allowed us to determine that changes in LPAR1/LPAR3 expression in HCC tissue were confined to a subset of cells located at the HCC-NTL margin. Further analysis of these LPAR1/LPAR3 positive cells revealed they also express progenitor/stem cell markers in the absence of hepatocyte markers. By screening established human hepatic tumor cells we determined the SKHep1 cell line exhibited a similar profile to the subset of cells that stain positive for both LPAR3 and cancer stem cell markers located at the HCC-NTL margin. Using SKHep1 cells *in vitro* we were able to conclude LPA stimulates cell migration in the SKHep1 cell line *via* an LPAR3-Gi-protein-MEK-ERK dependent mechanism, independent of Rho or PI3K-Akt signaling, both of which are present and activated following LPA stimulation of SKHep1 cells. Collectively these data provide detailed mechanistic evidence for a role for LPA-LPAR3 dependent signaling in a unique subset of cancer stem cells located at the tumor-NTL margin in HCC patients.

## RESULTS

### LPAR1 and LPAR3 expression is significantly increased in human HCC samples and localizes to the tumor margin

**Figure 1:**
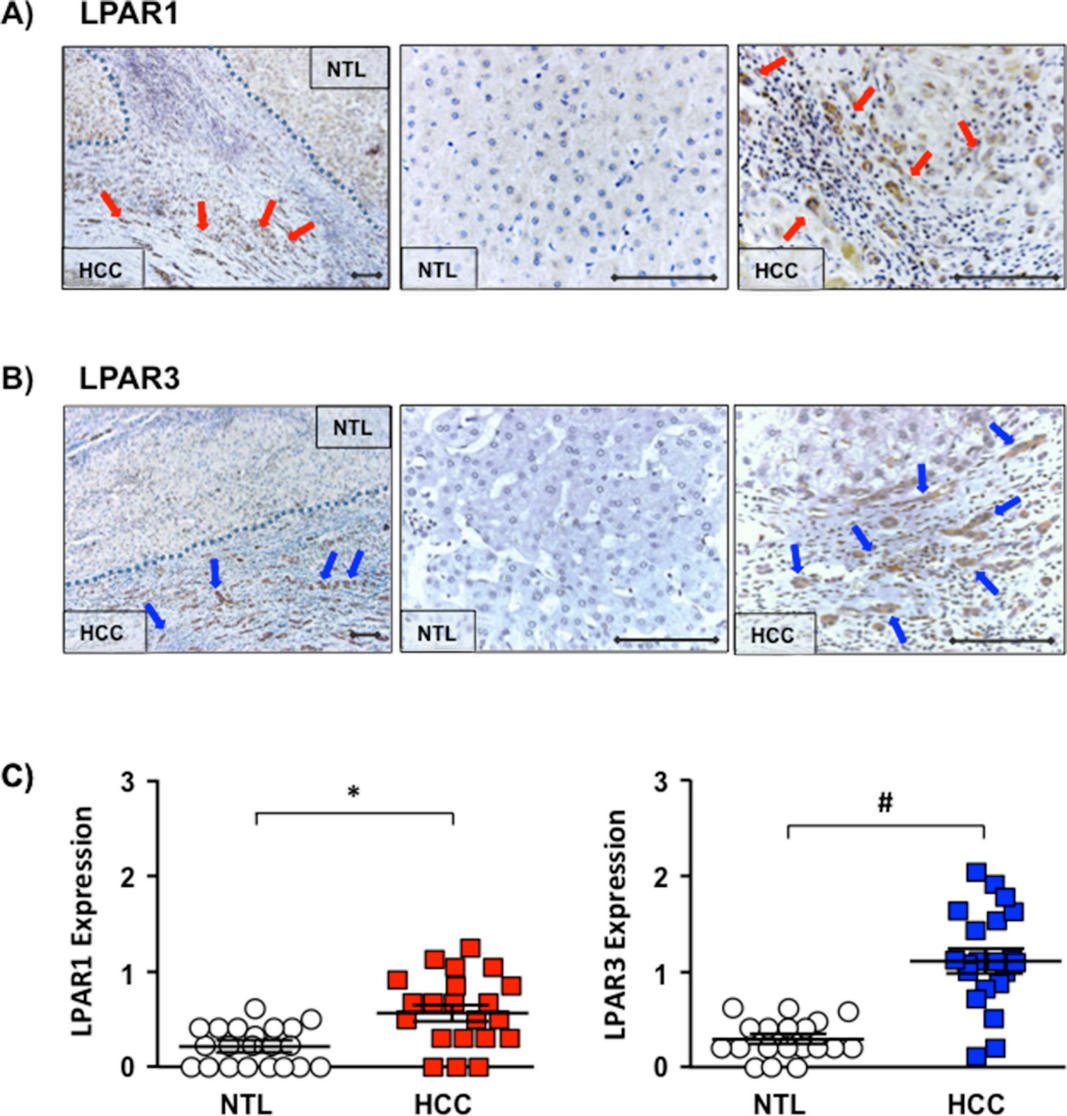
Increased LPAR1 and LPAR3 expression localized to the HCC-NTL margin **(A)** Representative immunohistochemical (IHC) images of LPAR1 expression in human hepatocellular carcinoma (HCC) tissue and the non-tumor liver (NTL) margin (x100 and x400 magnification), **♦**-**♦** = 100 μM), 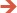 highlighted cells staining positive for LPAR1. **(B)** Representative IHC images of LPAR3 expression in human HCC-NTL tissue at x100 and x400 magnification (**♦**-**♦** = 100 μm), 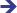 highlighted cells staining positive for LPAR 3. **(C)** Quantification of LPAR1 expression in NTL (white circles) and HCC (red squares) (*n =* 21) and LPAR3 expression in NTL (white circles) and HCC (blue squares) (*n =* 19). Each point represents an average score (0–4) from 10 random fields/HCC or NTL tissue/patient. *p < 0.05 and *p < 0.001 HCC vs. NTL).

Immunohistochemical (IHC) analysis was performed on archived human HCC samples from patients with varying underlying etiologies (*Supplementary Table 1*) using antibodies specific against LPAR1 or LPAR3 (Figure 1A and 1B, *Supplementary Figure S1*). Using this approach we demonstrated significantly increased LPAR1 expression in HCC *vs.* NTL (Figure 1C, IHC score 0.58 ± 0.08 *vs.* 0.21 ± 0.04; HCC *vs.* NTL; *p < 0.05). Overall, LPAR1 expression was increased in 71% of patients (15/21) and was most apparent at the NTL-HCC margin (Figure 1A). Analysis of LPAR3 also demonstrated significantly increased expression in HCC *vs.* NTL (Figure 1C, IHC score 1.13 ± 0.12 *vs.* 0.28 ± 0.05, HCC *vs.* NTL, *p < 0.001). Of note, increased LPAR3 in HCC was more pronounced than that observed for LPAR1 and occurred in 89% of patients (17/19), the most significant expression again being localized to the HCC-NTL margin (Figure 1B).

### SKHep1 cells express LPAR1 and LPAR3

**Figure 2:**
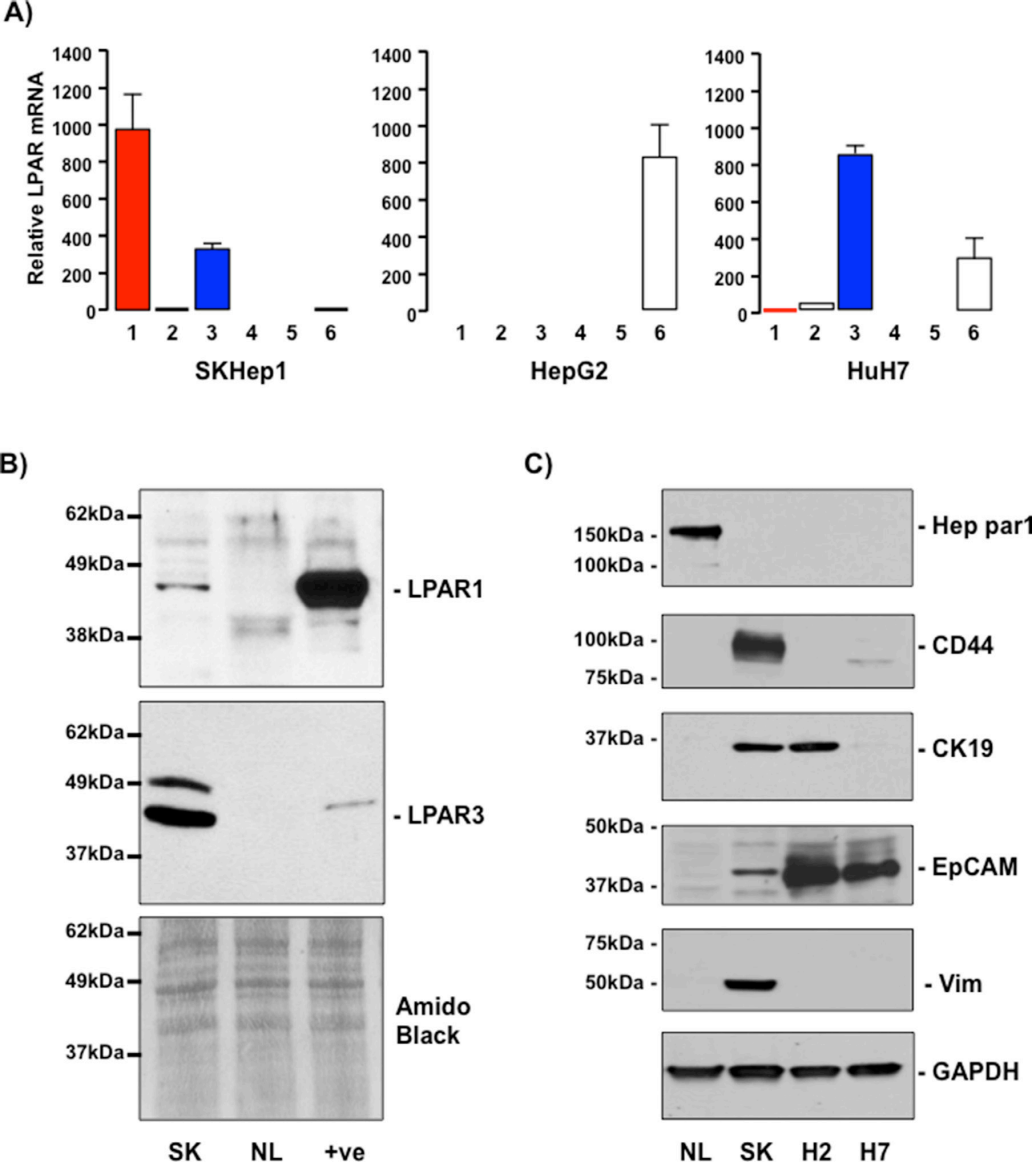
SKHep1 cells express LPAR1 and LPAR3 and mesenchymal/cancer stem cell markers **(A)** Relative mRNA expression of LPARs1–6 in SKHep1, HepG2 and HuH7 cells. Expression is calculated relative to β2-microglobulin expression (2^−ΔCt^ × 10^4^). **(B)** LPAR1 and LPAR3 protein expression in lysates prepared from SKHep1 cells (SK), normal liver from non-HCC burdened patients (NL). Positive control (+ve) for LPAR1 was rat brain lysate and PC3 human prostate cancer cell lysate for LPAR3. Membranes were stained with amido black as an indicator of total protein loading. **(C)** Representative Western blots of lysates prepared from normal liver (NL), SKHep1 (SK), HepG2 (H2) and HuH7 (H7) cells probed with antibodies against Hep par1, CD44, CK19, EpCAM or vimentin (Vim). Membranes were stripped and probed with GAPDH as a house-keeping protein/loading control.

LPAR1–6 mRNA expression was examined in 3 human tumor cell lines of hepatic origin (SKHep1, HepG2, and HuH7). These data demonstrate the LPAR mRNA profile of SKHep1 cells resembled the *in vivo* LPAR profile detected in cells localized to the HCC-NTL margin; SKHep1 cells expressing LPAR1 and LPAR3 mRNA (Figure 2A). Conversely, LPAR6 mRNA was readily detectable in HepG2 and HuH7 cells but not SKHep1 cells, and mRNAs for LPARS2, 4 and 5 were barely detectable, if at all, in any of the cell lines (Figure 2A). LPAR protein was measured by Western blot and LPAR1 and LPAR3 were detected in SKHep1 cells but not in normal liver from non-HCC patients (NL) (Figure 2B).

### SKHep1 cells and LPAR3 positive cells in the HCC-NTL margin express stem cell but not hepatocyte markers

**Figure 3:**
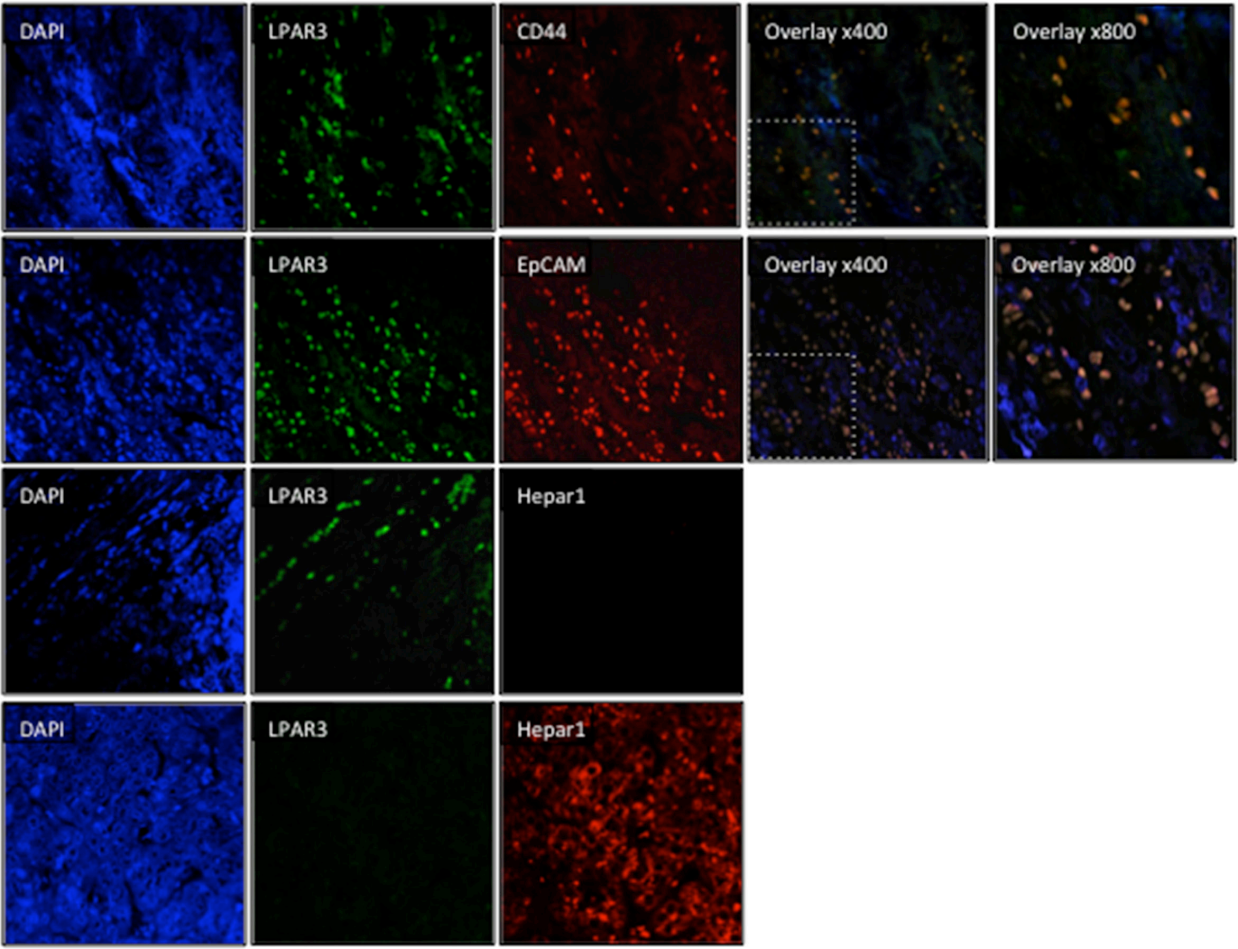
LPAR3 expression colocalizes with stem cell markers at the tumor-liver margin Representative immunofluorescent histochemistry images of human HCC-NTL sample margins stained with DAPI (nuclear stain [blue]) or antibodies selective against LPAR3 (green), stem cell or progenitor markers (CD44 or EpCAM; red), or Hep par1, a hepatocyte specific marker (red). Red and green images were merged to define co-localization of LPAR3 with other markers (orange).

The SKHep1 cell line is a human tumor cell line that lacks hepatic parenchymal cell (hepatocyte) markers that was originally derived from the ascites fluid of a patient presenting with an adenocarcinoma [29]. Recently, Eun *et al*. reported extensive expression of mesenchymal and cancer stem cell markers in SKHep1 cells [30]. These findings led us to examine the expression of Hep par 1 (an hepatocyte marker [31]), CD44, Cytokeratin-19 (CK-19), epithelial cell adhesion molecule (EpCAM), and vimentin (markers of mesenchymal stem cells, hepatic tumor/progenitor cells, and cells of mesenchymal origin [32–35]). By Western blot analyses we demonstrate NL readily expresses Hep par 1 in the absence of CD44, CK-19, EpCAM or vimentin (Figure 2C). Analysis of human cell lines revealed only SKHep1 expresses CD44 and vimentin (Figure 2C), while EpCAM was most readily detectable in HepG2 and HuH7 cells and CK19 was expressed in SKHep1 and HepG2 cells (Figure 2C). In light of these *in vitro* data sections from human HCC-NTL margin were then analyzed by immunofluorescent histochemistry (IFHC) using antibodies against LPAR3 and EpCAM, CD44, or Hep par1. These data demonstrated extensive co-localization of LPAR3 with CD44 and EpCAM in the HCC-NTL margin (Figure 3) in the absence of LPAR3-Hep par1 co-localization (Figure 3).

### Exogenous LPA stimulates SKHep1 cell motility

**Figure 4:**
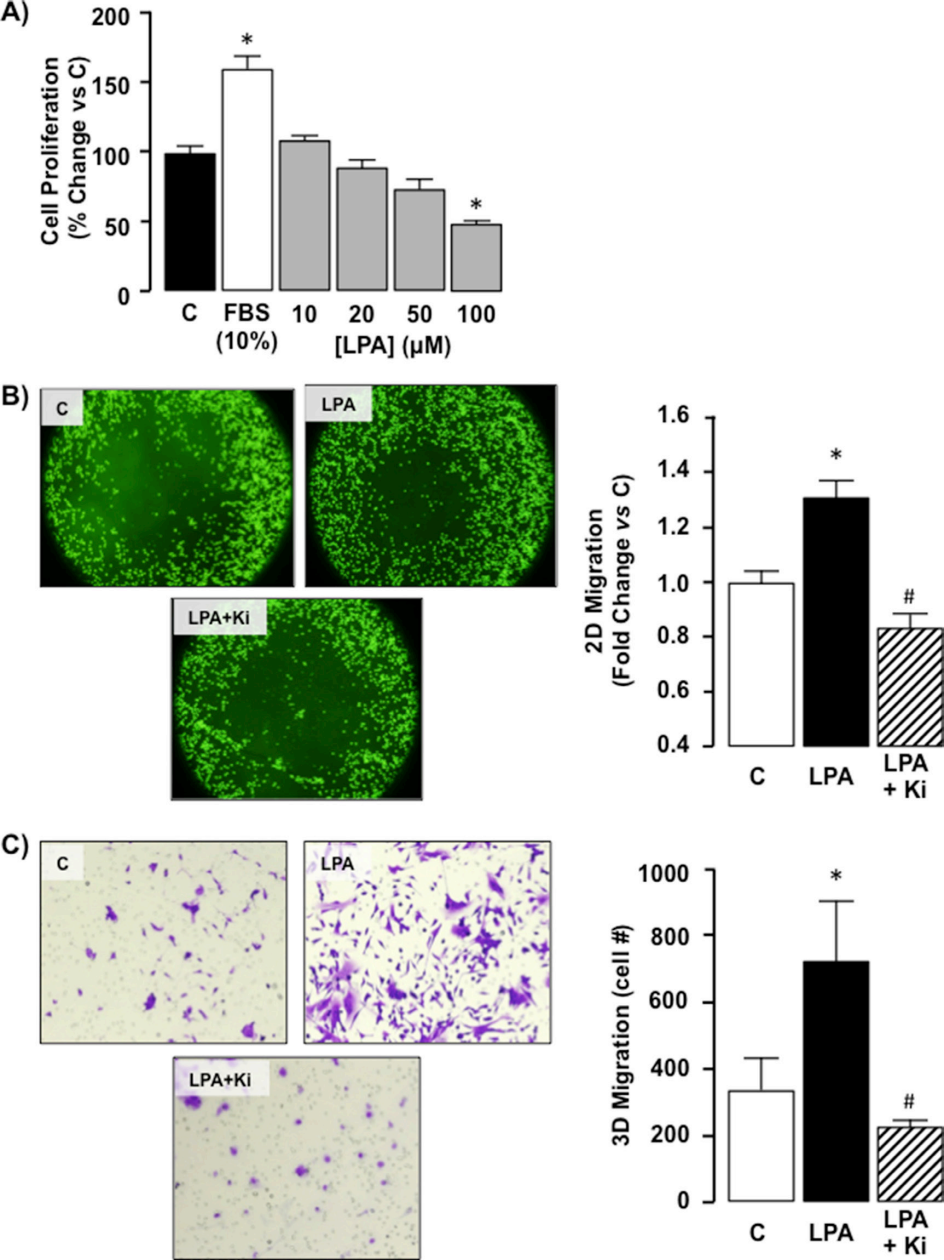
Exogenous LPA does not affect proliferation but does stimulate migration in SKHep1 cells via LPAR1/LPAR3 receptors (**A**) Cell proliferation was measured in quiescent cells maintained in serum depleted (0.1% FBS) medium (C). Following serum stimulation (10% FBS) or LPA treatment (0–100 μM) cell counts were performed and expressed as% change *vs*. C. *p < 0.05 LPA *vs*. C. (**B**) Representative images of 2D cell migration for SKHep1 cells in the absence of LPA (control; (**C**) following LPA stimulation (LPA, 10 μM overnight), and following LPA treatment in the presence of an LPAR1–3 antagonist (Ki16425 (Ki), LPA + Ki, 10 μM 24 h prior to LPA). Cell migration toward the (unseeded) center was measured using relative fluorescent units normalized to C. *n =* 3 independent experiments, 8-well replicates, *p < 0.05 LPA *vs*. C, *p < 0.05 LPA *vs*. LPA+Ki. (**C**) Representative images of 3D cell migration for SKHep1 cells in the absence of LPA (C), following LPA stimulation (10 μM, overnight), and following LPA treatment in the presence of Ki (LPA + Ki, 10 μM, 24 hrs prior to LPA). SKHep1 cells were stained with crystal violet and manually counted for 5 random fields/insert, *n =* 3 independent experiments, *p < 0.05 LPA *vs* C, *p < 0.05 LPA *vs.* LPA+Ki.

We next examined the effect of exogenous LPA on SKHep1 cell function. Analysis of cell proliferation demonstrated LPA did not significantly affect SKHep1 growth in serum-depleted medium (SDM; 0.1% FBS) at LPA levels ≤ 50 μM. At LPA levels ≥ 100 μM cell numbers were significantly lower than SDM alone (Control) suggesting potential LPA toxicity at these higher doses (Figure 4A, *p < 0.05 100 μM LPA *vs*. Control).

We next assessed the effect of exogenous LPA on SKHep1 motility *in vitro*. Using a 2D migration assay we demonstrated LPA significantly increased SKHep1 migration toward the unseeded area of the plate (Figure 4B, *p < 0.05 LPA vs. Control). Concurrently SKHep1 cells were pre-treated with the LPAR1–3 inhibitor Ki16425 (Ki; 10 μM) [36] followed by LPA. Using this approach Ki abolished the effect of LPA on cell migration (Figure 4B, *p < 0.05 LPA *vs*. Ki + LPA). Parallel experiments were performed to determine 3D cell migration in responses to LPA (10 μM) using a Transwell insert assay. Using this approach LPA significantly increased SKHep1 cells actively migrating through the membrane (Figure 4C, *p < 0.05 LPA *vs*. Control). Pretreatment of SKHep1 cells with Ki (10 μM) abolished LPA-mediated 3D migration (Figure 4C, *p < 0.05 LPA *vs*. Ki + LPA).

### Exogenous LPA stimulates G_12/13−_ and Gi-dependent intracellular signaling in SKHep1 cells

**Figure 5:**
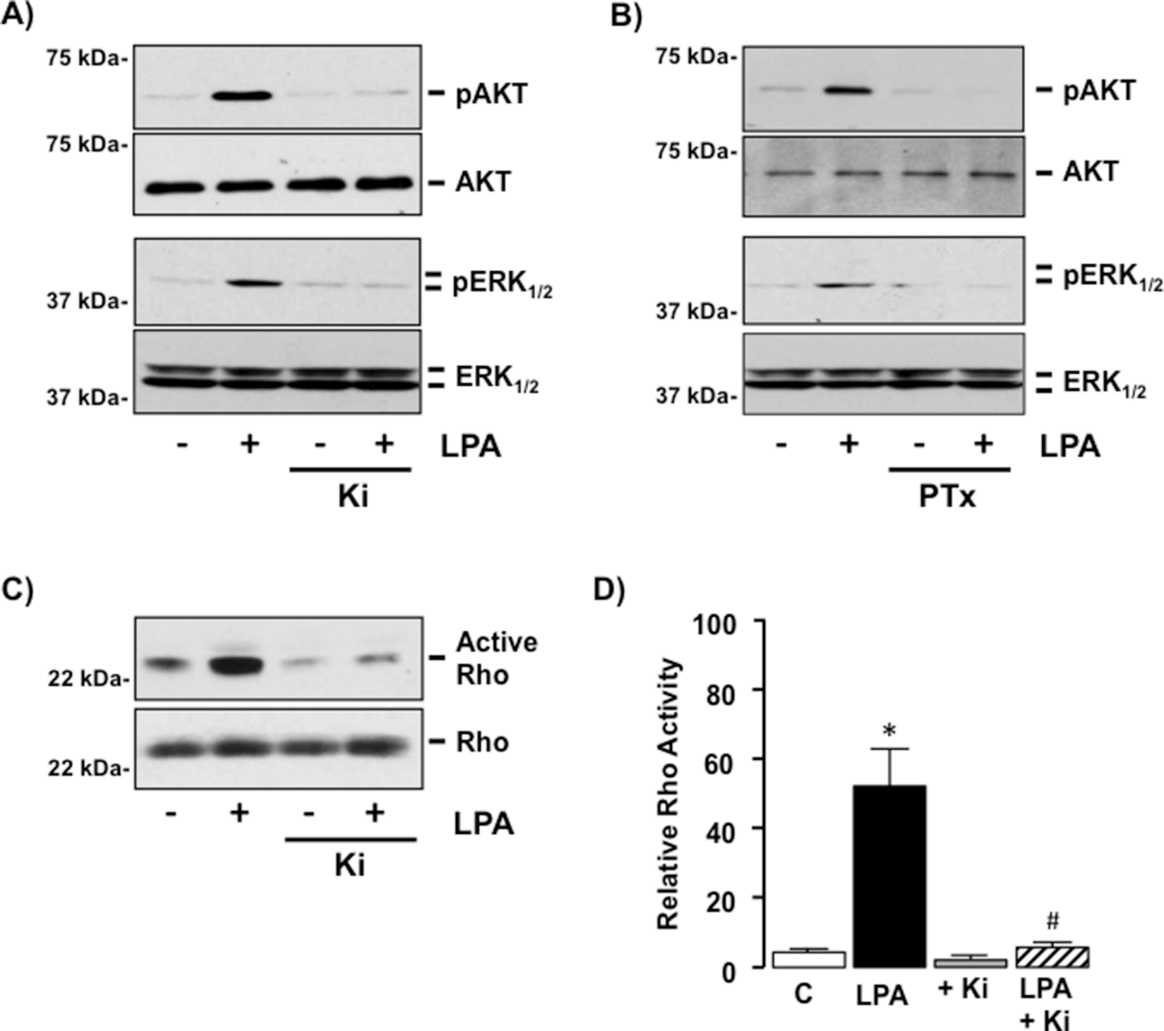
LPA regulates G-protein-dependent intracellular signaling in SKHep1 cells via LPAR1 and/or 3 *in vitro* **(A)** Representative Western blots demonstrating total Akt/pAkt in cell lysates from SKHep1 cells in the absence or presence (−/+) of LPA (10 μM), −/+ pretreatment (24 hrs) with the LPAR1–3 antagonist Ki16425 (Ki, 10 μM). **(B)** Representative Western blots demonstrating total ERK_1/2_/pERK_1/2_ in cell lysates from SKHep1 cells −/+ LPA (10 μM), −/+ pretreatment (24 hrs) with the Gi-protein antagonist pertussis toxin (PTx, 100 nM). **(C)** Rho-activity measured in cell lysates from SKHep1 cells −/+ LPA (10 μM), −/+ pretreatment with Ki (24 hrs) assessed using an active Rho pull-down assay. **(D)** Rho-activity measured in cell lysates from SKHep1 cells −/+ LPA (10 μM), −/+ pretreatment with Ki (24 hrs) assessed using a G-LISA-RhoA Activation assay, *n =* minimum of 3 experiments performed in triplicate. *p < 0.05 LPA *vs.* untreated control (C), *p < 0.05 LPA *vs.* LPA + Ki.

To determine the intracellular pathways regulated in SKHep1 following LPA-LPAR binding we measured the activity of intracellular signaling pathways regulated by Gi-protein and G_12/13−_ protein signaling (pAKT/pERK_1/2_ and Rho respectively). This approach demonstrated LPA (10 μM) stimulated AKT, ERK_1/2_ and Rho activity (Figure 5A–5D). Pre-treatment of cells with Ki or pertussis toxin (PTx, Gi-protein inhibitor; 100 nM) [37] abolished the effect of LPA on AKT and ERK_1/2_ activation (Figure 5A and 5B) while inhibition with Ki abrogated the effects of LPA on Rho activation (Figure 5C and 5D, *p < 0.05 LPA *vs*. Ki + LPA).

### LPA-stimulated cell migration is Gi-protein dependent in SKHep1 cells

**Figure 6:**
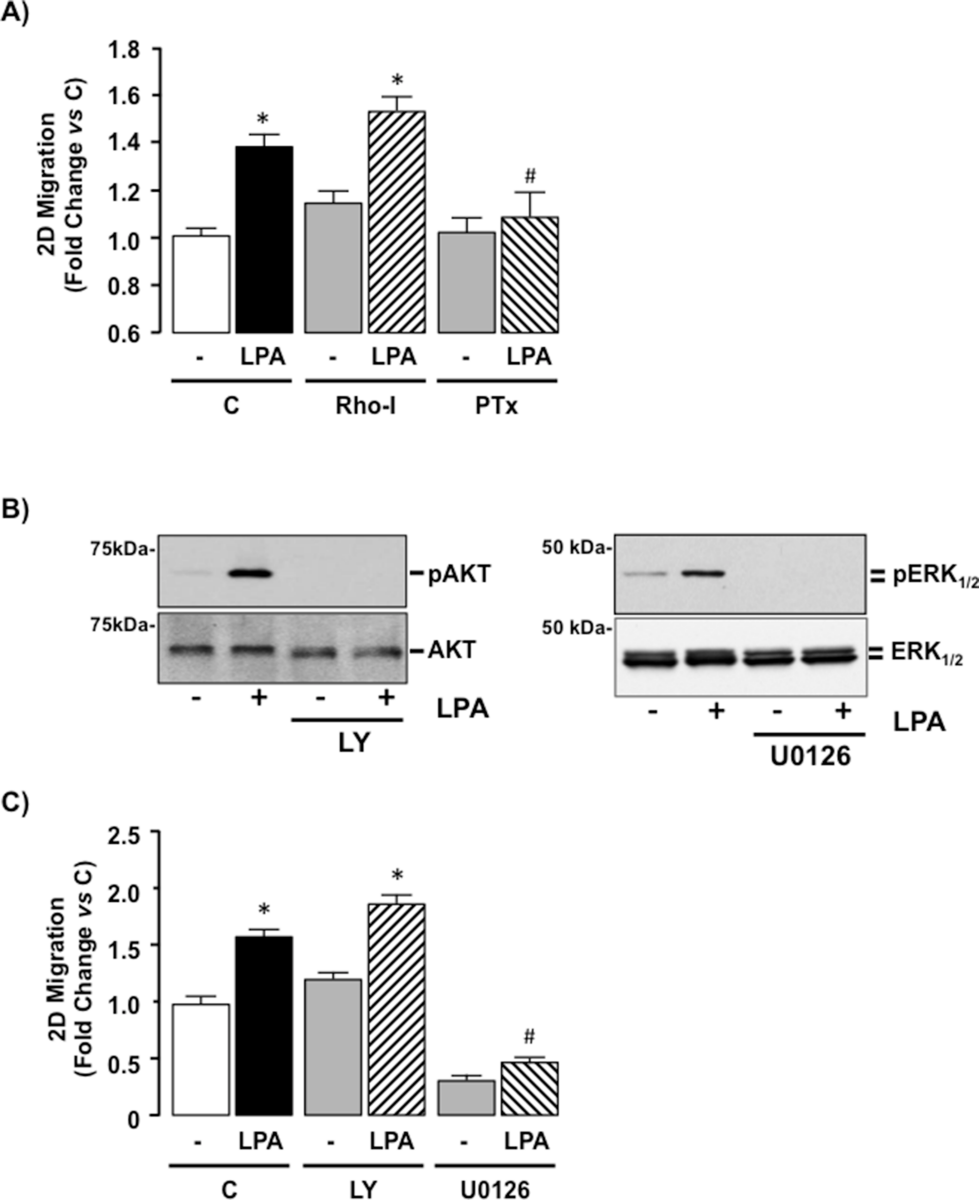
LPA-mediated cell migration is Gi-protein dependent in SKHep1 cells *in vitro* (**A**) SKHep1 cells were seeded for 2D migration assays and pretreated (24 hrs) with vehicle (C), a Rho-inhibitor (Rho-I, 1 μg/mL) or a Gi-protein inhibitor (pertussis toxin [PTx], 100 nM) followed by addition of vehicle (−) or LPA (10 μM, overnight). Cell migration was measured and normalized to untreated control. *p < 0.05 LPA *vs*. pair-matched non-LPA-treated, *p < 0.05 LPA *vs*. LPA+PTx. (**B**) Representative Western blots demonstrating total Akt/pAkt and total ERK_1/2_/pERK_1/2_ in cell lysates prepared from SKHep1 cells in the absence (−) or presence (+) of LPA (10 μM) without or following pretreatment with a PI3K inhibitor (LY294002 [LY], 40 μM) or a MEK inhibitor U0126 (5 μM). (**C**) Effect of LPA (10 μM) on 2D SKHep1 cell migration in the absence (C) or presence of LY or U0126. *p < 0.05 LPA *vs*. pair-matched non-LPA-treated, *p < 0.05 LPA *vs*. LPA+U0126.

To determine the contribution of intracellular signaling pathways on cell function SKHep1 cells were treated with LPA (10 μM) in the absence or presence of a Rho-inhibitor (Rho-I; 1 μg/mL) [38] or PTx (100 nM) and 2D cell migration measured. Rho-I failed to significantly alter SKHep1 responsiveness to LPA (Figure 6A, *p < 0.05 Rho-I vs. Rho-I + LPA, and *Supplementary Figure S2*). Conversely PTx abolished LPA-mediated 2D migration in SKHep1 cells (Figure 6A, and *Supplementary Figure S2*, *p < 0.05 LPA *vs*. PTx + LPA). Because Gi-proteins signal *via* both α-subunit and βγ–dimer-dependent mechanisms, we pre-treated SKHep1 cells with either a PI3K inhibitor (LY294002 [LY], 40 μM) [39] or a MEK-ERK_1/2_ inhibitor (U0126, 5 μM) [40]. Using this approach we demonstrated effective inhibition of LPA-dependent PI3K and MEK-ERK_1/2_ activity in the presence of LY and U0126 respectively (Figure 6B). Inhibition of PI3K signaling (LY) failed to significantly alter the effect of LPA on 2D migration (Figure 6C and *Supplementary Figure S2*, *p < 0.05 LPA *vs*. LY + LY), whereas inhibition of MEK-ERK_1/2_ signaling abolished LPA-dependent 2D migration (Figure 6C, *p < 0.05 LPA *vs*. U0126 + LPA, and *Supplementary Figure S2*). Cell viability was confirmed by performing a Neutral red uptake assay (data not shown).

### LPAR3 mediates Gi-ERK-dependent migration in response to LPA in SKHep1 cells

**Figure 7:**
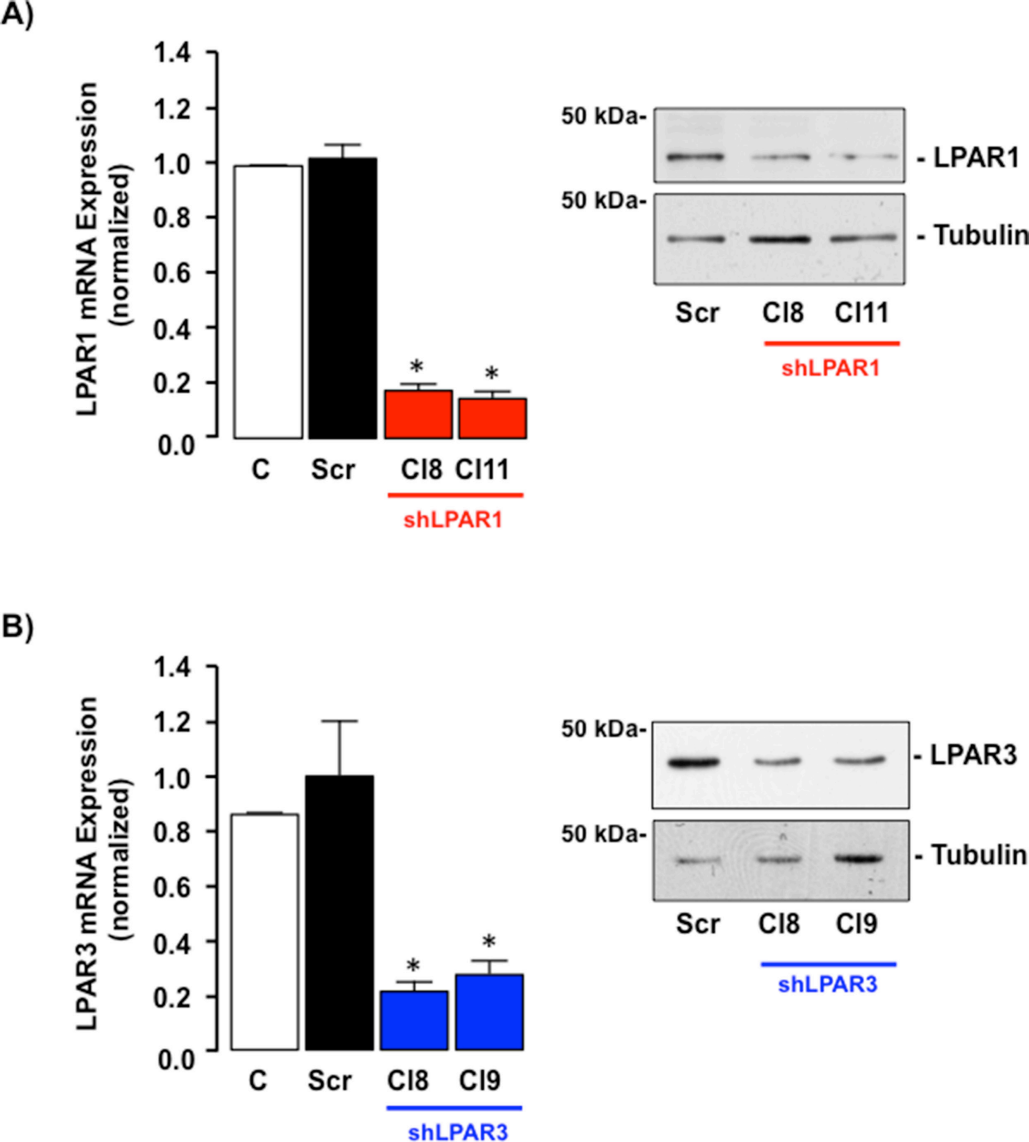
LPAR1 or LPAR3 is effectively knocked down in SKHep1 cells *in vitro* using stable shRNA transfection (**A**) qRT-PCR mRNA and representative Western blot protein analysis of LPAR1 expression in non-transfected SKHep1 cells or SKHep1 clones following stable transfection with an off-target scrambled sequence (Scr) or an shRNA against LPAR1 (shLPAR1 Cl8 and Cl11). *p < 0.05 Scr *vs.* shLPAR1. (**B**) qRT-PCR mRNA and representative Western blot protein analysis of LPAR3 expression in non-transfected SKHep1 cells or SKHep1 clones following stable transfection with an off-target scrambled sequence (Scr) or an shRNA against LPAR3 (shLPAR3 Cl8 and Cl9). *p < 0.05 Scr *vs*. shLPAR3.

SKHep1 cell lines with down regulated LPAR1 or LPAR3 expression were created by stable transfection with shRNA constructs. From these (a minimum of) two clones were selected in which target LPAR mRNA was reduced by ≥ 70% (Figure 7A and 7B, *p < 0.05 *vs.* untransfected (C) or cells transfected with scrambled sequence (Scr)). Clones were expanded and decreased target protein expression confirmed by Western blot (Figure 7A and 7B). Inhibition of target LPARs did not significantly alter expression of other LPAR isoforms (data not shown).

**Figure 8:**
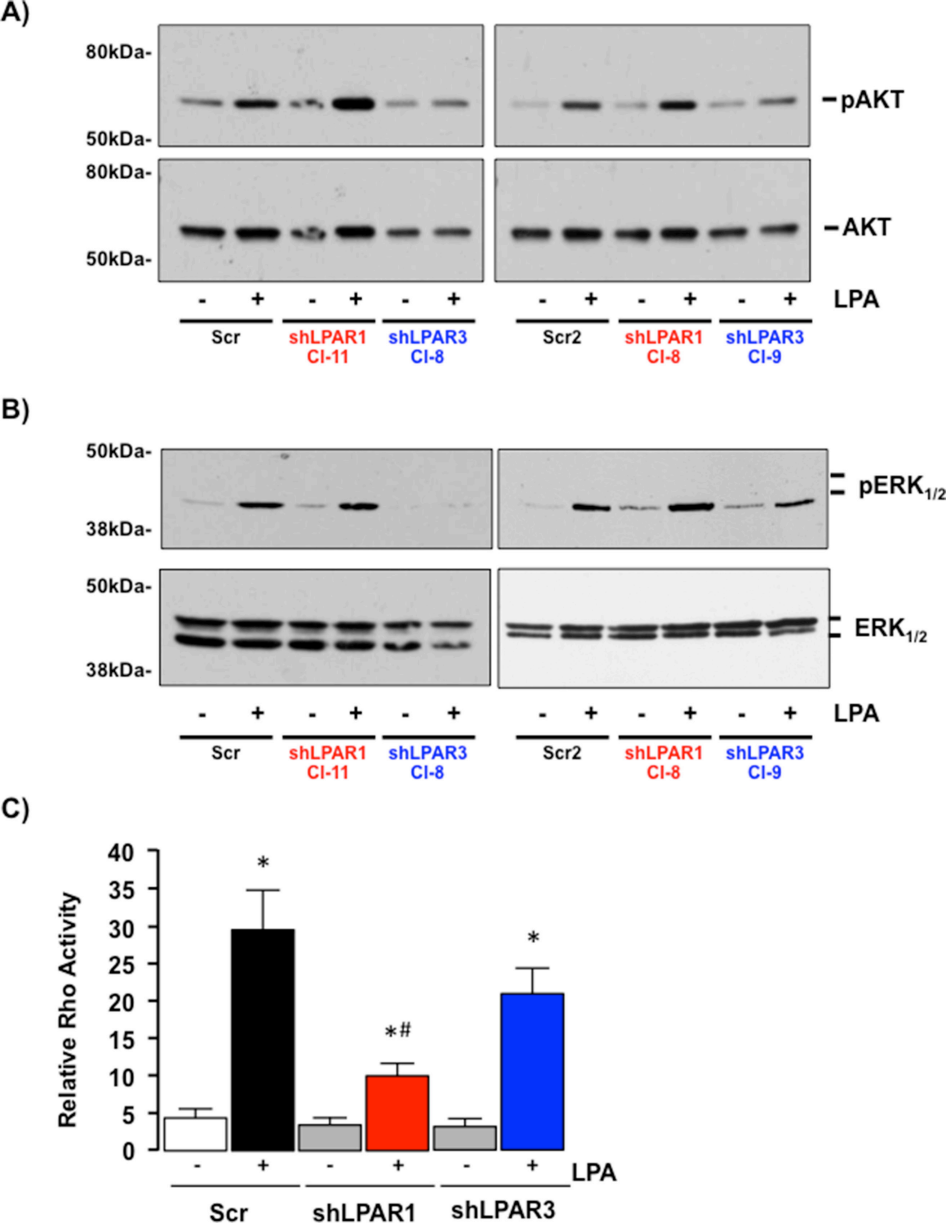
LPAR1 and LPAR3 regulate different intracellular signaling cascades in SKHep1 cells (**A**) Representative Western blots demonstrating total Akt/pAkt in cell lysates prepared from SKHep1 cells stably transfected with an off-target scrambled sequence (Scr) or an shRNA against LPAR1 (shLPAR1 clones 11 and 8 (Cl-11/Cl-8)) or LPAR3 (shLPAR3, clones 8 and 9 (Cl-8/Cl-9) in the absence (−) or presence (+) of LPA (10 μM). (**B**) Representative Western blots demonstrating total ERK_1/2_/pERK_1/2_ in cell lysates prepared from SKHep1 cells stably transfected with an off-target scrambled sequence (Scr) or an shRNA against LPAR1 (shLPAR1 clones 11 and 8 (Cl-11/Cl-8)) or LPAR3 (shLPAR3, clones 8 and 9 (Cl-8/Cl-9) in the absence (−) or presence (+) of LPA (10 μM). (**C**) Rho activity in SKHep1 clones stably transfected with an off-target scrambled sequence (Scr) or an shRNA against LPAR1 (shLPAR1) or LPAR3 (shLPAR3) in the absence (−) or presence (+) of LPA (10 μM). *n =* 3 independent experiments performed in duplicate, *p < 0.05 LPA *vs*. pair-matched non-LPA-treated, *p < 0.05 shLPAR1 + LPA *vs*. Scr + LPA and shLPAR3 + LPA.

Inhibition of LPAR1 failed to significantly alter the effect of LPA on either AKT or ERK_1/2_-MAPK activity (Figure 8A and 8B) but significantly blunted the effect of LPA on Rho activation (Figure 8C, *p < 0.05 LPA *vs.* Control [Scr, shLPAR1 and shLPAR3], *p < 0.05 shLPAR1 + LPA *vs*. Scr + LPA). In contrast, inhibition of LPAR3 significantly inhibited LPA-dependent AKT and ERK_1/2_ activation (Figure 8A and 8B) without significantly altering Rho activity (Figure 8C, *p < 0.05 shLPAR3 + LPA *vs*. Control [shLPAR3 and scr]).

**Figure 9:**
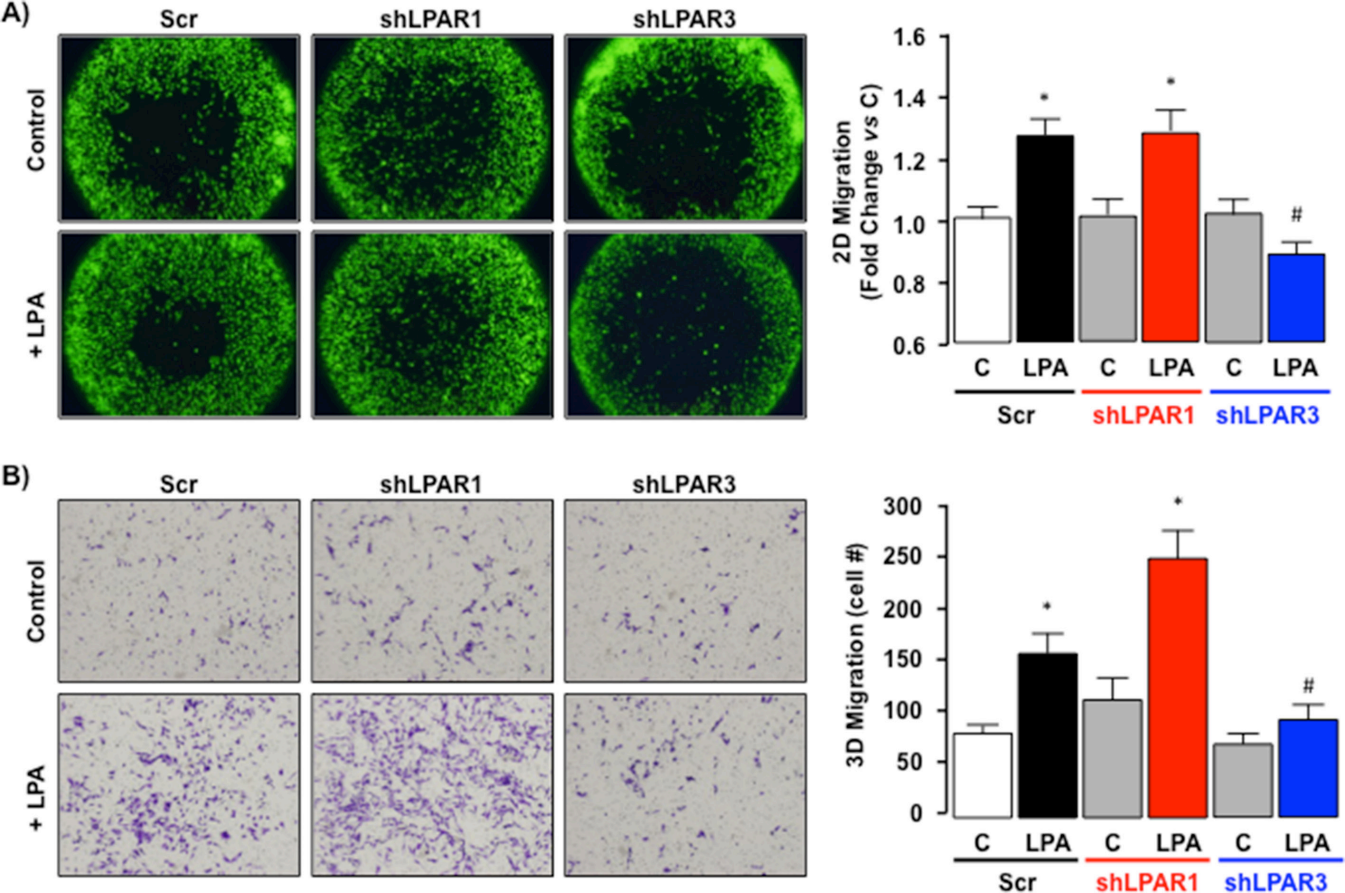
LPA-dependent migration signals via LPAR3 not LPAR1 in SKHep1 cells *in vitro* **(A)** Representative images of 2D cell migration for SKHep1 clones stably transfected with an off-target scrambled sequence (Scr) or an shRNA against LPAR1 (shLPAR1; data shown are for clone 8) or LPAR3 (shLPAR3; data shown are for clone 8) in the absence (Control (C)) or presence (+) of LPA (10 μM). Data from ≥ 2 independent clones repeated in 8 replicates were collected and expressed as fold change vs. pair-matched non-LPA-treated control (C). *p < 0.05 C *vs*. LPA, *p < 0.05 shLPAR3 + LPA *vs*. Scr + LPA and shLPAR1 + LPA. **(B)** Representative images of 3D cell migration for SKHep1 clones stably transfected with an off-target scrambled sequence (Scr) or an shRNA against LPAR1 (shLPAR1; data shown are for clone 11) or LPAR3 (shLPAR3; data shown are for clone 9) in the absence (Control (C)) or presence (+) of LPA (10 μM). Data from ≥ 2 independent clones repeated in triplicate were collected and expressed as change *vs*. pair-matched non-LPA-treated control (C) or Scrambled sequence non-LPA-treated control. *p < 0.05 C *vs*. LPA, *p < 0.05 shLPAR3 + LPA *vs*. Scr + LPA and shLPAR1 + LPA.

Down-regulating LPAR1 expression in SKHep1 cells did not significantly alter LPA-induced 2D or 3D migration (Figure 9, *p < 0.05 shLPAR1 + LPA *vs*. shLPAR1). Conversely, inhibition of LPAR3 expression effectively blocked LPA-stimulated 2D and 3D migration (Figure 9, *p < 0.05 shLPAR3 + LPA vs. Scr + LPA).

## DISCUSSION

In this study we report the microenvironment in human hepatocellular carcinoma (HCC) and non-tumor liver (NTL) is characterized by a subset of cells expressing LPAR1 and LPAR3, a profile shared by the human hepatic tumor-derived SKHep1 cell line. Stimulating SKHep1 cells with LPA *in vitro* led to G-protein-dependent activation of Rho (*via* G_12/13_-protein signaling) and Akt/ERK-MAPK (*via* Gi-protein signaling) resulting in cell migration in the absence of changes in proliferation. Using an shRNA approach we demonstrate LPA-mediated migration in SKHep1 cells is dependent on LPA-LPAR3 signaling *via* a Gi-ERK-MAPK signaling cascade. Conversely, while LPA-LPAR1 signaling led to activation of a Rho-dependent signaling cascade, inhibiting this pathway failed to alter LPA-dependent SKHep1 migration. Finally, re-examining human HCC revealed cells staining positive for LPAR3 in the HCC-NTL margin also stained positive for cancer stem cell (but not hepatocyte) markers, features shared by SKHep1 cells.

In the healthy liver LPAR1/LPAR3 are expressed at relatively low levels compared with other tissue types [4, 17–19]. Indeed, the relative absence of well-characterized LPAR subtypes (LPARs1–5) within the liver presents somewhat of a paradox in understanding how LPA affects liver function and hepatic pathogenesis. For example, in animal models, correlations between circulating LPA and degree of underlying liver injury are reported [41] yet LPARs 1–5 are barely detectable (if at all) in rodent hepatocytes/liver tissue [28]. Similarly, cirrhotic and HCC patients exhibit elevated serum LPA levels, while the relative expression of LPA species in patients at risk for developing HCC suggest serum LPA profiles may be beneficial in identifying those patients with HCC [10]. The characterization of the purinergic p2ry5 receptor as a novel non-EDG LPAR, LPAR6, and reports of LPAR6 expression in human and rodent liver, and human HCC tissue may help explain these findings. However, unlike NTL, HCC tissue, and other human hepatic tumor cell lines, we failed to detect significant LPAR6 expression in SKHep1 cells (relative to LPAR 1 and 3 mRNA). Additionally, we failed to detect the hepatocyte marker, Hep par1, in cells that stained for LPAR1/LPAR3 in human HCC tissue. These data suggest SKHep1 cells may not have originated as a result of hepatocyte transformation, and that LPAR6-dependent signaling is not a significant factor in mediating LPA-dependent activity in these cells.

Studies in other organ systems report LPA-LPAR1–3 signaling is involved in tissue fibrosis [42, 43]. Hepatic cirrhosis is the most common precursor for progression to HCC, and changes in LPA-LPAR1–3 signaling during hepatic stellate cell (HSC) activation is reported [44]. In addition to a role for LPA-LPAR1–3 signaling during hepatic fibroge nesis, studies by Mazzocca et al., report tumor-derived LPA increases peritumoral fibroblast recruitment, and subsequent transdifferentiation to myofibroblasts [20]. Myofibroblasts, in turn, play a crucial role in mediating the crosstalk between transformed hepatocytes and non-parenchymal, stromal liver cells [20, 45]. These findings suggest tumor-derived LPA can act as a paracrine signaling factor within the tumor microenvironment to promote tumor expansion by stimulating the accumulation and/or activity of myofibroblasts within the tumor [20]. We also demonstrated (the majority of) LPAR1/LPAR3 detected by IHC occurred at the NTL-HCC margin. Because we failed to detect significant LPAR1/LPAR3 expression within the larger tumor mass, or more distant NTL, and cells that stained for LPAR1/LPAR3 did not stain for the hepatocyte marker Hep par1, we hypothesized LPAR1/LPAR3 expression was confined to a non-HCC, non-hepatocyte cell population. While it remains possible this cell population is derived from HSCs (HCCs often being characterized by fibrous encapsulation [1]), the relative absence of staining for LPAR1/LPAR3 in NTL, even in cirrhotic patients, and the histologic appearance of LPAR1/LPAR3 positive cells led us to examine alternatives.

Several authors postulate the significance of hepatic stem cells during hepatocyte repopulation, hepatocyte transformation, and tumor progression [46–48]. Using antibodies against CD44 and EpCAM1 (markers of mesenchymal stem cells and liver cancer progenitor cells) we identified cells expressing LPAR3 in the HCC-NTL margin also expressed these stem cell markers. A review of the literature reveals SKHep1 cells, while widely reported and employed as *human HCC cells of mesenchymal origin*, do not express hepatic genes or exhibit endogenous liver cell (hepatocyte) functions [29], factors further supported by our failure to detect LPAR6 [27, 28]. Unlike many immortalized HCC cell lines, the SKHep1 line was not derived from a surgically resected liver tumor. Rather, the SKHep1 line was derived from ascites fluid of a patient with a hepatic adenocarcinoma, indicating a cell line with existing metastatic properties [29, 30, 49]. This evidence is further supported in a report by Eun *et al*., in which a detailed analysis of SKHep1 cells (using 22 different markers/antibodies) reports SKHep1 are characterized by classical mesenchymal stem cell markers in the absence of endothelial and hematopoietic markers. Furthermore, SKHep1 cells are able to differentiate to adipocytes and osteoblasts and form widespread, multi-organ site metastatic masses using an *in vivo*/SCID mouse model. Collectively Eun et al., conclude SKHep1 cells represent a transformation of mesenchymal stem cells into cancer stem cells [30].

Our data demonstrate that, in addition to a similar LPAR1/LPAR3 profile, the stem cell markers used on human tissue were also present in SKHep1 cells *in vitro*. These findings led us to address the effect and mechanisms of action by which LPA affects SKHep1 cells as a means to investigate the potential function of cells staining positive for LPAR3 in the HCC-NTL margin. All members of the LPAR family are G-protein coupled receptors and others report LPAR1 and 3 transduce signals *via* Gαi, Gαq and Gα_12/13_ (LPAR1) or Gαi and Gαq (LPAR3) subunits, the LPAR-Gα-protein interaction being dependent on factors that include cell type, agonist/LPA isoform, and the relative expression/interaction of the different LPAR subtypes within the cell [3, 4, 50]. Our studies demonstrate that intracellular signaling in SKHep1 cells in response to LPA is dependent on the LPAR subtype activated. Activation of LPAR1 stimulates Rho activity but LPA-LPAR1-Rho signaling does not mediate SKHep1 cell migration. Conversely, LPA-LPAR3 signaling stimulates PI3K and ERK-MAPK activity, *via* Gi-protein-dependent mechanism (s), and inhibition of Gi-ERK-MAPK abolished the effect of LPA on SKHep1 cell migration (effects mimicked by knocking down LPAR3 expression).

These data are both supportive and contraindicative to previous studies using tumor cell lines in culture, including the SKHep1 cell line. In a study by Park et al., LPA augmented SKHep1 invasion *via* LPAR1 signaling and matrix metalloproteinase 9 (MMP9). This conclusion is derived by the analysis of LPARs 1–3 mRNA expression and *in vitro* studies that stepwise analyze intracellular signaling mechanisms. Using this approach the authors determine that SKHep1 invasion following exogenous LPA requires PKCδ/p38 MAPK and PI3K/Akt activation, data that are initially mirrored by our findings. However, in their studies the authors report only LPAR1 mRNA, and not LPAR3 mRNA, expression in SKHep1 cells [51]. In contrast our data report readily detectable LPAR3 mRNA and protein in SKHep1. Furthermore, inhibition of LPAR1 or PI3K did not significantly alter LPA-mediated cell migration in our studies, whereas inhibition of LPAR3-Gi-protein-ERK-MAPK signaling abrogated LPA-dependent migration. Several reasons may explain such differences. At a fundamental level our group used different primers to measure LPAR3 mRNA expression. However, protein measurement by Western blot supported our LPAR3 mRNA expression data. Alternatively, an increasing body of literature addresses consistency within ‘banked’ cell lines, data that highlights the potential that many commonly employed cell lines exhibit differences due to prolonged propagation in culture [52]. Finally, there are inherent differences between the assays performed by Park *et al*., in which they measure invasion whereas as we measured migration [51].

While our data provides evidence that LPA stimulates cell movement in the SKHep1 cell line *via* an LPAR3-Gi-ERK MAPK pathway it also raises other intriguing questions. For example, we clearly demonstrate LPA stimulates Rho activity in SKHep1 cells and inhibition of LPAR1 expression indicates an LPA-LPAR1-Rho activation pathway. Studies using other cell lines, including cancer cells, report Rho is a significant factor in mediating a range of cell responses, including cell movement, and Rho is an important factor in determining the metastatic potential of cancer cells [53, 54]. Furthermore, several studies report LPAR1 is an abundantly expressed LPAR in a variety of cancers, including pancreas [15], colon, lung and breast [13], and Rho activation is mediated *via* Giα_12/13_ [55, 56], a Gα-protein associated with LPAR1 [3, 4, 57]. Conversely, ovarian cancer appears to be characterized by decreased LPAR1 expression [12] and tumor aggressiveness is associated with altered LPAR 2 and LPAR3, LPAR3 being capable of signaling *via* Gi-proteins [7, 58]. This appears to be mirrored in SKHep1 cells as inhibition of Gi-signaling (PTx) abolished the effects of LPA on migration. Interestingly, activation of Gi-proteins has the potential to exert dual effects *via* α-subunit (inhibition of adenylyl cyclase-cAMP-protein kinase A) and βγ-dimer (stimulation of MEK-ERK MAPK) activity [59]. Our data suggest LPA-LPAR3 dependent cell migration in SKHep1 cells is most likely to occur *via* a G-βγ-dimer dependent mechanism.

In conclusion, our data provide further evidence to support the notion that SKHep1 cells are not human HCC cells but rather, are a human cancer stem cell line. Furthermore, LPA stimulates migration (but not proliferation) in the SKHep1 cell line via LPAR3-Gi-protein-ERK-MAPK signaling. These data are particularly intriguing as a similar LPAR profile to that of SKHep1 cells exists in a sub-population of cells at the HCC-NTL margin, a cell population that also stains positive for stem cell, but not hepatocyte, markers. These data, along with previously published studies, suggest an important role for LPA-LPAR signaling within the tumor microenvironment whereby LPA is likely to act via either LPA-LPAR6 signaling in HCC cells, or via LPA-LPAR1/LPAR3 signaling in hepatic myofibroblasts and hepatic cancer progenitor cells. Given that healthy, non-parenchymal cells in the liver express LPAR1 and/or LPAR3 at low levels, this raises the possibility of developing selective therapies against LPAR1 and/or LPAR3 to slow or regress HCC progression.

## MATERIALS AND METHODS

### Institutional assurances and patient demographics

The Institutional Review Board (IRB) at Carolinas Medical Center granted approval to analyze surgical specimens of patients undergoing HCC resection. We obtained informed, written consent from patients for participation in the study and for collecting and storing their information for research purposes. A board certified pathologist confirmed HCC diagnosis and underlying pathology and patient demographics were recorded from 23 patients undergoing hepatic resection or transplant for HCC (*n =* 18 male: age 61.0 ± 11.4y, range 43–90y. *n =* 5 female: 70.6 ± 4.7y; range, 66–78y, *Supplementary Data Table 1*). Within this cohort 9/23 patients were HCV-positive, 5/23 were diagnosed with underlying non-alcoholic steatohepatitis (NASH), 2/23 with alcoholic steatohepatitis (ASH), 1/23 had autoimmune hepatitis, and 1/23 had HBV (*Supplementary Data Table 1*). Normal liver tissue from liver disease/tumor free patients (NL) was obtained from the NIH-sponsored Liver Tissue Cell Distribution System (LTCDS, Minneapolis, MN).

### Materials

Antibody sources/dilutions for the different procedures are provided in *Supplementary Table 2A*. A G-LISA Rho Activation Assay and total Rho ELISA kit to detect active/total Rho, and the Rho Inhibitor I (Rho-I) were purchased from Cytoskeleton Inc. (Denver, CO). Oleoyl-*sn*-3-glycerophosphate (LPA) and Ki16425 (Ki) were purchased from Cayman Chemical (Ann Arbor, MI). Pertussis toxin (PTx), U0126, and LY294002 (LY) were purchased from EMD Millipore (Billerica, MA).

### Histology and Immunohistochemistry (IHC)

Paraffin embedded human HCC with pair-matched, adjacent NTL tissue were obtained from the Department of Pathology (CMC), sectioned (5 μm), and H & E stained to allow HCC-NTL margin identification. Expression and localization of LPAR1 or LPAR3 were established by IHC as previously reported [27]. Ten random fields (x200 magnification) of (HCC-NTL) were blind-scored using a scale of 0–4 (0 = no detectable stain; 1 = 1–9% of cells stained; 2 = 10–19% cells stained; 3 = 20–29% cells stained; and 4 = >30% cells stained).

### Cell culture

Human SKHep1 and HepG2 cells (ATCC, Manassas, VA) and HuH7 cells (JCRB, Japan) were maintained in DMEM medium supplemented with 10% (*v/v*) fetal bovine serum (FBS) and antibiotics as previously reported [60].

### mRNA and protein expression

Relative mRNA expression was determined by quantitative real-time PCR (qRT-PCR) as previously reported [27] (primer sequences and reaction conditions are described in *Supplementary Table 2B*). Relative protein expression was determined by Western blot using whole cell lysates [27].

### Cell proliferation

Following cell plating (12-well plates; ≈ 90,000 cells/well) and attachment (18 Hrs), culture medium was replaced with SDM (0.1% (*v/v*) charcoal-stripped FBS (Gemini Bio-Products, Sacramento, CA) for 24 h followed by LPA treatment (0–100 μM, 24 h). At the end of this period cells were detached and cell proliferation measured by cell count-viability (trypan blue exclusion) analysis (Countess Automated Cell Counter, Life Technologies, Grand Island, NY) [60]. Data are means from a minimum of 4-wells per treatment group from ≥ 3 independent experiments.

### Cell migration

Two dimensional cell migration experiments were conducted using an Oris Cell Migration Assay (Platypus Technologies, Madison, WI). Briefly, SKHep1 cells were fluorescently stained (CellTracker Green, Life Technologies) and plated at 30,000 cells/well into 96-well plates equipped with rubber plugs in the absence or presence of inhibitors. After 24 hrs the plugs were removed, LPA added (10 μM final concentration) and cells incubated overnight. Migration was measured as relative fluorescence within the original plug “footprint”. Alternatively, cells were rinsed, fixed, stained with crystal violet, and images captured and analyzed for cell occupancy within the original unseeded plug “footprint” using the NIH ImageJ software. Data are means from a minimum of 4 independent experiments performed in 8-well replicates per treatment group.

Three-dimensional migration was measured using 8 μm pore Transwells (BD Biosciences, San Jose, CA) as previously reported. Briefly, cells were collected after 24 h in SDM (0.1% (*v/v*) charcoal-stripped FBS) and added to the plastic inserts of the Transwell chamber in the absence or presence of inhibitors and 0.7 ml SDM, with or without LPA (10 μM), was added to the lower chamber. Following overnight incubation the inserts were removed, rinsed in phosphate buffered saline (PBS), and cells wiped from the internal surface. Cells on the outer surface were fixed in methanol, stained with crystal violet, rinsed in distilled water, and 5 random fields counted/experimental condition. Data are from a minimum of three independent experiments/group.

### Intracellular signaling activity

Cells were placed in SDM for 24 h then treated with LPA (10 μM) or vehicle. Cells were harvested, lysed and resultant total/active protein expression detected by Western blot [27].

### Rho activity

Rho activation was measured by Western blot analysis following pull-down of active Rho with an Active-Rho pull-down/detection kit as per the manufacturer's instructions (Thermo-Fisher Scientific, Waltham, MA) and compared to total Rho. Rho activity was also measured using a G-LISA RhoA Activation Kit and a Total RhoA ELISA as per the manufacturer's instructions (Cytoskeleton Inc.).

### LPAR1 and 3 knock-down

SKHep1 cells were stably transfected with plasmids (EDG-2 shRNA plasmid (h) [Cat # sc-43746-SH] and EDG-7 shRNA plasmid (h) [cat # sc-37088-SH], Santa Cruz Biotechnology Inc., (Dallas TX)) possessing short hairpin (sh) RNA (shRNA sequences) against either LPAR1 or LPAR3 respectively, or an arbitrary off-target sh sequence using Lipofectamin 2000 (Life Technologies). 48 h after transfection Puromycin was added (1 μg/ml final concentration) and selection held for 12 d. Positive clones were picked up, expanded and analyzed for LPAR expression by qRT-PCR and Western blot.

### Statistical analysis

Data (where appropriate) are presented as mean ± SEM. Statistical analysis was performed using GraphPad software (La Jolla, CA). Pair-wise combinations within a group were analyzed by Student's *t*-test. *p* < 0.05 was considered significant.

## SUPPLEMENTARY FIGURES AND TABLES


