# Supplementary material for: Expression and function of lysophosphatidic acid receptors (LPARs) 1 and 3 in human hepatic cancer progenitor cells
Source: Oncotarget. 2015 Dec 20;7(3):2951–67. doi: 10.18632/oncotarget.6696 (PMC4823083; doi:10.18632/oncotarget.6696)
Supplement: Supplementary file 1 [file oncotarget-07-2951-s001.pdf]

Expression and function of lysophosphatidic acid receptors (LPARs) 1 and 3 in human hepatic cancer progenitor cells

Supplementary Materials

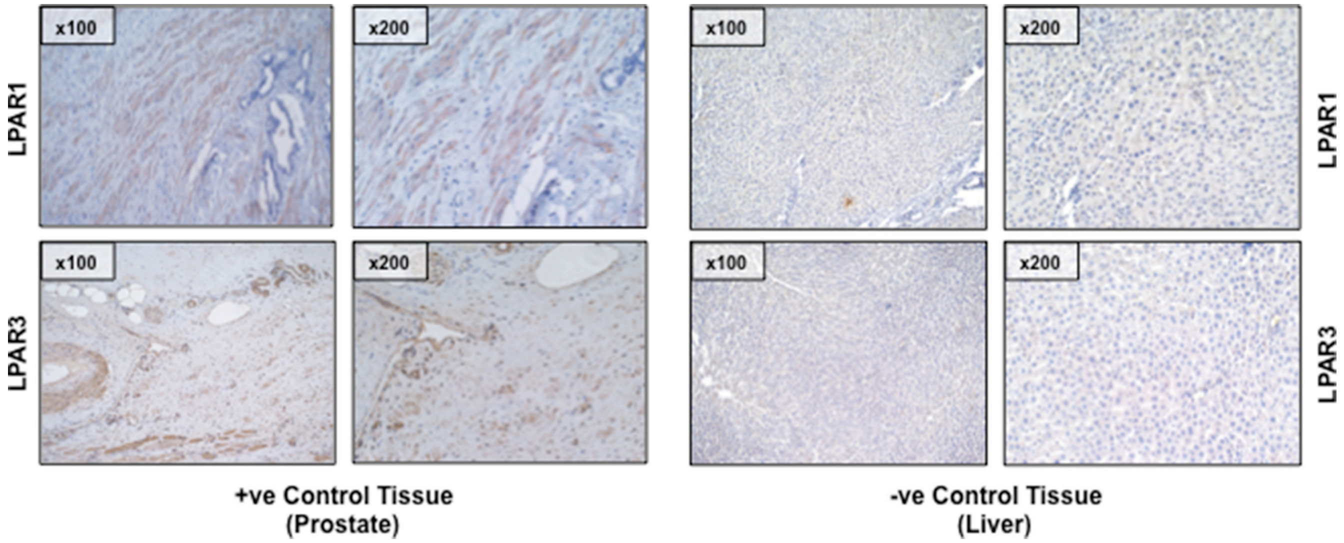

Supplementary Figure S1: Representative immunohistochemical (IHC) images of LPAR1 and LPAR3 protein expression in positive control (+ve) prostate tissue and negative control (−ve) liver tissue from a non-HCC burdened patient at ×100 and ×200 magnification.

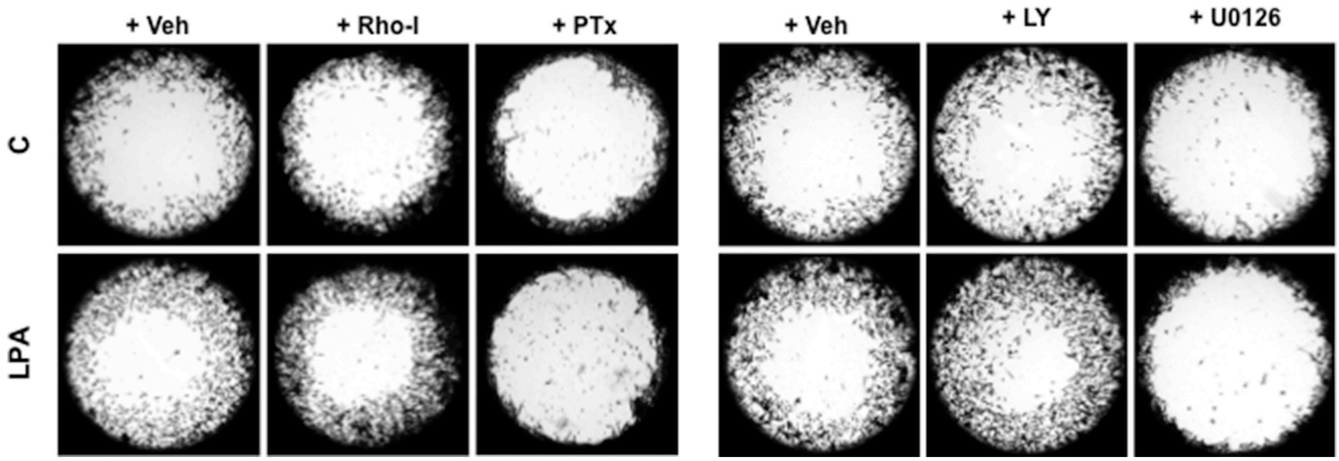

Supplementary Figure S2: Representative images of 2D cell migration for SKHep1 cells in the absence of LPA (control; C) or following LPA stimulation (LPA, 10 µM, overnight) in the presence or absence of vehicle (Veh) a Rho inhibitor I (Rho-I, 1 µg/ml), pertussis toxin (PTx, 100 nM), a PI3K inhibitor (LY294002 [LY], 40 mM) or a MEK-ERK$_{1/2}$ inhibitor (U0126; 5 µM). Cell migration toward the (unseeded) center of the field was measured (cumulative data are presented in Figure 6A and 5C).

Supplementary Table S1: Patient demographics and clinical parameters

ID#	Sex	Age	BMI	Race	Tumor Size (cm)	Focality	Etiology	Differentiation	Cirrhosis	LPAR3 Score HCC	LPAR3 Score NTL
**HCXX865**	M	61	24	W	4.0	Single	HCV	Moderate	Yes	2.0	0.4
**HCXX308**	M	64	26	Other	1.5	Single	HCV	Moderate	Yes	0.7	0.2
**HCXX324**	M	49	26	AA	1.8	Single	HCV	Well	Yes	1.5	0.2
**HCXX451**	M	51	23	W	2.7	Single	HCV	Well	Yes	0.1	0.0
**HCXX792**	M	58	28	W	3.0	Single	HCV	Well	Yes	0.2	0.2
**HCXX076**	M	57	28	W	3.0 × 1.3	Multi	HCV	Moderate	Yes	1.0	0.2
**HCXX399**	M	66	20	W	3.0–5.0	Multi	HCV	Moderate	Yes	1.6	0.6
**HCXX258**	M	55	n/a	AA	7.0	Single	HCV	Poor	Yes	1.2	0.0
**HCXX540**	M	47	28	AA	1.5	Single	HCV	Moderate	Yes	1.2	n/a
**NAXX885**	M	76	29	W	3.0–4.0	Multi-	NASH	Moderate	Yes	1.5	n/a
**NAXX786**	M	61	55	W	13.0	Single	NASH	Moderate	No	0.5	0.4
**NAXX622**	M	68	45	W	1.0 × 9.0	Multi	NASH	Moderate-Poor	No	0.8	0.4
**NAXX710**	F	71	23	W	2.0	Single	NASH	Moderate	Yes	1.4	0.0
**NAXX355**	M	67	28	W	0.5 × 2.0	Multi	NASH	Moderate	Yes	0.2	1.1
**ASXX713**	M	73	25	W	3.0	Single	ASH	Moderate	Yes (mild)	1.9	0.2
**ASXX624**	M	54	21	W	6.5	Single	ASH	Moderate	No	1.1	0.4
**AIXX599**	F	66	23	W	0.2–2.0	Multi	AIH	Moderate-Well	No	1.6	0.4
**HBXX352**	F	71	20	As	3.8	Multi	HBV	Poor		1.0	0.2
**OtXX244**	M	58	29	AA	2.3	Single	n/a	Well	Yes	1.1	0.6
**OtXX229**	M	43	20	W	3.5	Single	Fibro-lamellar	Well	No	1.0	0.2
**OtXX568**	M	90	33	W	4.9	Single	HX-ve	Moderate	No	1.1	0.0
**OtXX562**	F	78	32	W	2.5 × 7.0	Multi	n/a	Moderate	Yes (mild)	1.6	0.6
**OtXX353**	F	67	38	W	8.7	Single	Mild fatty liver	Poor	Yes	1.3	0.3

W = white; AA = African American, As = Asian; HCV = viral hepatitis C; HBV = viral hepatitis B; NASH = non-alcoholic steatohepatitis; ASH = alcoholic steatohepatitis; AIH = autoimmune hepatitis; N/A = Data not available.

Supplementary Table S2A: Antibodies employed

Antibody	Source	Use	Dilution
**LPAR1**	Novus Biologicals (Littleton, CO)	IHC	1:100
**LPAR3**	EMD Millipore (Billerica, MA)	IHC	1:200
**LPAR1**	Novus Biologicals (Littleton, CO)	WB	1:500
**LPAR3**	Sigma (St Louis, MO)	WB	1:500
**Pan-Akt (11E7)**	Cell Signaling Technologies (Danver, MA)	WB	1:1000
**Phospo-AKT (pAkt) (ser473)**	Cell Signaling Technologies (Danver, MA)	WB	1:2000
**ERK1/2**	Santa Cruz Biotechnology, (Dallas, TX)	WB	1:500
**Phospho-p44/42 MAPK (Tyr204/ Tyr187)**	Cell Signaling Technologies (Danver, MA)	WB	1:1000
**LPAR3**	EMD Millipore (Billerica, MA)	IFHC	1:200
**CD44**	Novus Biologicals (Littleton, CO)	IFHC	1:250
**EpCAM**	Novus Biologicals (Littleton, CO)	IFHC	1:200
**Hepar1**	Dako (Carpinteria, CA)	IFHC	1:50
**CD44**	Novus Biologicals (Littleton, CO)	WB	1:500
**EpCAM**	Novus Biologicals (Littleton, CO)	WB	1:250
**Hep par1**	Dako(Carpinteria, CA)	WB	1:400

IHC = Immunohistochemistry; WB = Western blot; IFHC = Immunofluorescent histochemistry

Supplementary Table S2B: Primers used for qRT-PCR

Target	Forward	Reverse
LPAR1	5′ ATGGCTGCCATCTCTACTTCCATCC 3′	5′ CCA TTC TGT GGC AAG ATG CTT TCC 3′
LPAR2	5′ ACGCTCAGCCTGGTCAAGAC3′	5′ AAC CAT CCA GGA GCA GTA CCA C 3′
LPAR3	5′ ACT ACC TGT TGG CTA ATT TAG CTG CTG C 3′	5′ GGG AAG CAG TCA AGC TAC TGT CCA GA 3′
LPAR4	5′ GGG TGA CAG AAG ATT CAT TGA CTT CC 3′	5′ GCA GTT CCA GAG ATC TTG CAG AGG 3′
LPAR5	5′ ATG TTA GCC AAC AGC TCC TCA ACC 3′	5′ GCC AGT GGT GCA GTG CGT AGT A 3′
LPAR6	5′ TAT GGT AAG CGT TAA CAG CTC CCA 3′	5′ GGA CTT TGA GGA CGC AGA TGA AA 3′
β2M	5′ GCT GTG CTC GCG CTA CTC TCT CTT TC 3′	5′ TCG GAT GGA TGA AAC CCA GAC ACA 3′

β2M = β-2-microglobulin (house keeping gene). qRT-PCR conditions: 2 µg of total RNA was reverse transcribed with High Capacity Reverse Transcription Kit (Applied Biosystems); ~50 ng of resulting cDNA was taken for qRT-PCR in 20 µl reaction with SsoFast EvaGreen Supermix (Bio-Rad). Amplification was done with CFX-Connect (Bio-Rad) instrument, thermal profile was initial denaturation (95° for 30 s) followed by 39 cycles (5 s at 95°, and 10 s at 56°C)
